# The Dark Side of Fibroblasts: Cancer-Associated Fibroblasts as Mediators of Immunosuppression in the Tumor Microenvironment

**DOI:** 10.3389/fimmu.2019.01835

**Published:** 2019-08-02

**Authors:** Lea Monteran, Neta Erez

**Affiliations:** Department of Pathology, Sackler School of Medicine, Tel Aviv University, Tel Aviv, Israel

**Keywords:** CAFs, immunosuppression, immune modulation, inflammation, tumor microenvironment

## Abstract

Cancer-associated fibroblasts (CAFs) are prominent components of the microenvironment in most types of solid tumors, and were shown to facilitate cancer progression by supporting tumor cell growth, extracellular matrix remodeling, promoting angiogenesis, and by mediating tumor-promoting inflammation. In addition to an inflammatory microenvironment, tumors are characterized by immune evasion and an immunosuppressive milieu. In recent years, CAFs are emerging as central players in immune regulation that shapes the tumor microenvironment. CAFs contribute to immune escape of tumors via multiple mechanisms, including secretion of multiple cytokines and chemokines and reciprocal interactions that mediate the recruitment and functional differentiation of innate and adaptive immune cells. Moreover, CAFs directly abrogate the function of cytotoxic lymphocytes, thus inhibiting killing of tumor cells. In this review, we focus on recent advancements in our understanding of how CAFs drive the recruitment and functional fate of tumor-infiltrating immune cells toward an immunosuppressive microenvironment, and provide outlook on future therapeutic implications that may lead to integration of preclinical findings into the design of novel combination strategies, aimed at impairing the tumor-supportive function of CAFs.

## Introduction

Tumors are complex multicellular systems, characterized by reciprocal interactions between cancer cells and the tumor microenvironment (TME). The non-cancerous components that comprise the TME are central to all stages of tumorigenesis, progression, and metastasis (1). The TME is composed of the extracellular matrix (ECM), as well as various cell types including immune cells, endothelial cells, pericytes, and fibroblasts.

In non-cancerous homeostatic conditions, resident tissue fibroblasts are important sentinels of tissue integrity (2). Fibroblasts can sense and respond to mechanical changes, as well as to various tissue damage signals and react by differentiating to myofibroblasts that orchestrate tissue repair and wound healing, mediated by their ECM synthesis and remodeling and by their crosstalk with innate immune cells (3–5). Dysregulation of the physiological wound healing response and chronicity of inflammatory responses lead to fibrosis and scarring, characterized by excess ECM production and deposition by activated fibroblasts. These physiological functions of tissue fibroblasts are hijacked in cancer-associated fibroblasts (CAFs) in the microenvironment of tumors, consistent with the description of tumors as “wounds that do not heal” (6).

Cancer-associated fibroblasts (CAFs) are a vastly heterogeneous stromal cell population and are prominent components of the microenvironment in solid tumors. In some cancer types, including breast and pancreatic carcinomas, CAFs are the most prominent stromal cell type. The presence and function of activated CAFs in the microenvironment are associated with worse prognosis in multiple cancers (7). Moreover, tumors with high stromal signatures have been found to be associated with therapy resistance and disease relapse (8, 9).

CAFs are composed of multiple subpopulations that were shown to have diverse origins, including reprogrammed resident tissue fibroblasts (10, 11), bone marrow-derived mesenchymal cells (MSCs) (12, 13), adipocytes (14), and endothelial cells (15). Functionally, CAFs were shown to enhance tumor growth by several mechanisms: directly promoting cancer cell proliferation via secretion of growth factors, by inducing angiogenesis and by remodeling the ECM, which supports tumor cell invasion (5, 10, 16–18). Importantly, CAFs were also implicated in mediating tumor-promoting inflammation in various cancer types via secretion of cytokines and chemokines that mediate the recruitment and activation of immune cells, and by their reciprocal interactions with immune cells in the TME (2, 19). Studies in recent years have elucidated that this plethora of tumor-promoting activities of CAFs is mediated by functionally distinct subpopulations of fibroblasts (12, 20–22). Analysis of CAFs at the single cell level in the coming years will undoubtedly add complexity to the emerging landscape of CAF functional heterogeneity.

The role of the immune system in cancer is multi-faceted: In addition to an inflammatory microenvironment, tumors are also characterized by immune evasion and an immunosuppressive milieu, that were acknowledged as hallmarks of cancer (23). In order to survive and proliferate in the primary tumor site and in distant organs, which may be initially hostile, tumor cells must escape immune surveillance and avoid killing by cytotoxic lymphocytes. This is achieved by shaping the immune microenvironment toward a tolerant and immunosuppressive milieu, characterized by the presence of immature myeloid cells, T regulatory cells, decreased levels of infiltrating killer cells (T cells and NK cells), and dysfunction of their cytotoxic activity (24, 25). These mechanisms of immune escape and suppression are achieved by tumor cell downregulation of antigen presentation, elevated expression of surface inhibitory molecules, and secretion of immunosuppressive factors (24).

In addition to tumor cell-mediated signaling that drives immune suppression, fibroblasts are emerging as central players in shaping the TME toward an immunosuppressive and growth-promoting phenotype (26). CAFs contribute to immune escape via upregulation of immunosuppressive cytokine production and immune checkpoint ligands, exclusion of anti-tumor CD8^+^ T cells from cancer cells, and by affecting the functional differentiation of tumor infiltrating inflammatory cells.

In this review, we focus on recent advancements in our understanding of how CAFs affect the recruitment and functional fate of tumor-infiltrating immune cells toward shaping an immunosuppressive tumor microenvironment, and examine future therapeutic implications.

## CAFs Orchestrate Recruitment of Immune Cells

### Recruitment of Myeloid Cells

Myeloid cells are the most abundant hematopoietic cells in the body and are critical components of the tumor microenvironment, that contribute to all aspects of tumor progression (1). Myeloid cells in the TME include various populations of tumor-associated macrophages (TAMs), neutrophils, eosinophils, basophils, dendritic cells (DC) and mast cells (1, 27, 28). In addition, immature myeloid cells that express CD11b, Ly6G and/or Ly6C are sometimes referred to as myeloid-derived suppressor cells (MDSCs), based on their ability to functionally suppress the proliferation and activity of T cells. MDSCs are characterized by their expression of Arginase (ARG1), TGF-β, Programmed death-ligand 1 (PD-L1)/ 2, IL-10, Prostaglandin E2 (PGE2), S100A8/A9 and Indoleamine-pyrrole 2,3-dioxygenase (IDO), and by their capacity to regulate dendritic and T cell functions. MDSCs are commonly divided into two subsets based on their expression of surface markers: monocytic MDSCs (CD11b^+^Ly6C^high^Ly6G^−^), and granulocytic (or polymorphonuclear) MDSCs (CD11b^+^Ly6C^int^Ly6G^high^) (29, 30). Notably, these surface markers are used to identify mouse MDSCs. Human tumor-associated MDSCs are identified by their expression of CD33^+^CD14^+^HLA-DR^low/−^ (monocytic MDSCs) or CD11b^+^CD14^−^CD15^+^/CD66b^+^ (29).

CAFs were shown to recruit macrophages into the TME in multiple mouse models of cancer, including squamous cell, prostate and breast carcinomas (19, 31, 32). In a mouse model of spontaneous lymphoma, tumor-educated CAFs (derived from MSCs) recruited CD11b^+^Ly6C^+^ monocytes, F4/80^+^ macrophages, and CD11b^+^Ly6G^+^ neutrophils via the CCL2–CCR2 axis, thus enhancing tumor growth (33). Moreover, in a mouse model of breast cancer lung metastasis, MSCs that were pre-conditioned with TNFα and co-injected with tumor cells were shown to recruit CXCR2^+^ neutrophils by secreting CXCR2 ligands (CXCL1, CXCL2, and CXCL5), resulting in enhanced lung metastasis (34). Expression of chemoattractants for myeloid cells was suggested to be mediated by enhanced expression of miR-1246 in breast cancer CAFs, in an NF-κB dependent manner (35). Notably, recent understanding of CAF heterogeneity implicates a distinct subpopulation of inflammatory CAFs (iCAFs) rather than myofibroblast-like CAFs (myCAFs) in the induction and maintenance of an inflammatory milieu via their expression of inflammatory mediators (IL-6, IL-11, CXCL1, CXCL2) (21, 36). While these findings were described in a mouse model of pancreatic cancer, they likely represent a general phenomenon whereby specific functions of CAFs in affecting immune cells are mediated by distinct subpopulations (12, 37).

Recruitment of tumor-promoting myeloid cells by CAFs is associated with shaping their functional differentiation toward an immunosuppressive phenotype: Secretion of Chitinase-like protein 3 (Chi3L1) by mammary CAFs was shown to drive an M2-like phenotype in recruited macrophages, associated with reduced infiltration of CD8^+^ T lymphocytes (19). Interestingly, the expression of fibroblast activation protein (FAP) in CAFs in multiple cancer types was shown to be associated with recruitment of immunosuppressive cells: In a mouse model of hepatic cancer, a subset of FAP^+^ fibroblasts had an inflammatory phenotype directed by STAT3 activation and increased CCL2 expression, resulting in enhanced recruitment of CCR2-expressing circulating MDSCs and enhanced tumor growth (38).

Importantly, recruitment of myeloid cells by CAFs was also shown to be associated with resistance to therapy: In a mouse model of transplantable colorectal carcinoma (CRC), FAP^high^ fibroblasts were found to recruit myeloid cells via CCL2, leading to resistance to anti-PD-1 immune checkpoint therapy which was abrogated by targeting FAP. These findings were validated in human CRC tissue sections, where the abundance of FAP^high^ fibroblasts was in correlation with increased infiltration of myeloid cells and inversely correlated with infiltrated T cells (39). Similarly, pharmacological targeting of FAP in a transplantable model of pancreatic cancer resulted in decreased macrophage recruitment and enhanced T cell infiltration (40). Moreover, targeting of FAP^+^ fibroblasts by immunization with an adenoviral vector in both transgenic and transplantable mouse models of melanoma abrogated the recruitment and function of immunosuppressive cells including monocytic and polymorphonuclear MDSCs within the TME (41).

CCL2-mediated recruitment of circulating monocytes by CAFs was also demonstrated in models of breast cancer, *in vivo* and in a 3D *ex-vivo* model (42, 43). Notably, while recruitment of macrophages into tumors by CAFs is operative in various cancer types, the molecular pathways are distinct: In primary *in vitro* cultures, CAFs isolated from human prostate tumors were found to recruit monocytes by secreting stromal cell-derived factor 1 (SDF1)/CXCL12. Moreover, these SDF1-producing CAFs enhanced M2-like polarization of circulating monocytes, reflected by high production of the immune suppressive cytokine IL-10 (44). These findings agree with the demonstrated functional role of CAF-derived SDF1 in promoting tumor growth and immunosuppression (45, 46).

Recruitment of myeloid cells into tumors by CAFs is not limited to monocytes: platelet-derived growth factor receptor A (PDGFRα)^+^ CAFs isolated from murine tumors were shown to be a major source of the granulocytic chemoattractant CXCL1, and to mediate the accumulation of Ly6C^−^Ly6G^+^ granulocytic cells (granulocytic MDSCs) with potent immune-suppressive activity, assessed by their ability to suppress T cell proliferation. Interestingly, this pathway may be an adaptive response to anti-CSF1R therapy, as it was induced in CAFs following treatment with CSF1R inhibitor in models of colon, lung, breast carcinomas and melanoma (47). These findings instructed the design of combination therapy, to block CSF1R signaling as well as CAFs: Combining CSF1R inhibitor with a CXCR2 antagonist blocked granulocyte infiltration and resulted in strong delay in tumor growth in models of lung carcinoma and melanoma (47). Interestingly, mast cells were also shown to be recruited by CAFs: CAFs isolated from hormone-dependent prostate tumors mediated the recruitment of CXCR4-expressing mast cells by secreting CXCL12 (48).

One of the suggested mechanisms for CAF-mediated recruitment of myeloid cells to the TME is the expression of a senescence-associated secretory phenotype (SASP) gene signature. Cellular senescence was originally thought to be a tumor-suppressive mechanism that limits malignant transformation by arresting cell proliferation. However, studies in recent years have shown that senescent fibroblasts acquire a senescence-associated secretory phenotype (SASP) that supports their pro-inflammatory and tumor-promoting functions (49, 50). Moreover, the acquisition of a senescent phenotype by CAFs was shown to contribute to recruitment of immunosuppressive cells: In a mouse model of stromal-specific induced senescence, senescent dermal fibroblasts were shown to mediate the formation of an immunosuppressive microenvironment by enhancing the recruitment of CD11b^+^Ly6C^−^Ly6G^high^ cells and T regulatory (CD3^+^CD4^+^FOXP3^+^) cells, and enhanced ECM deposition. Co-injection of senescent dermal fibroblasts with squamous cell carcinoma cells demonstrated that SASP-induced shaping of the immune microenvironment promotes tumor growth. SASP-mediated tumor promotion was inhibited by targeting SASP-derived IL-6 or by depleting Ly6G^+^ cells (51).

**Figure 1:**
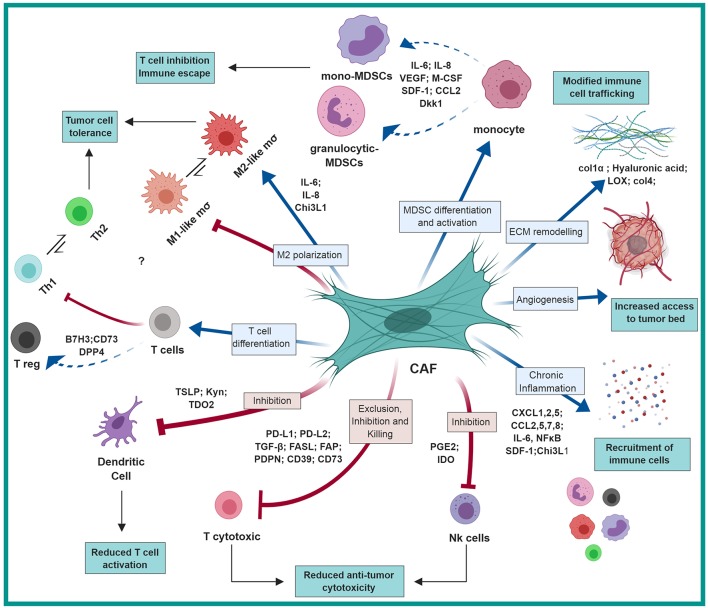
CAF-mediated immunosuppression: CAFs shape the immune microenvironment in tumors toward a pro-tumorigenic and immunosuppressive milieu by affecting the recruitment and function of various innate and adaptive immune cells. Red arrows represent negative regulation/inhibition and blue arrows represent positive regulation/induction. This figure was designed by using graphical elements from BioRender.

Thus, by employing multiple molecular pathways, CAFs recruit myeloid cells into tumors, that contribute to the formation of an immunosuppressive immune milieu (Figure 1).

### Recruitment of Regulatory T Cells

CAFs were found to potentiate the recruitment, differentiation and survival of T regulatory cells, contributing to the formation and maintenance of an immunosuppressive microenvironment. Treg cells are immunosuppressive T lymphocytes characterized by their expression of the IL-2 receptor α-chain (CD25) and the transcription factor forkhead box P3 (FOXP3). The mechanisms by which Treg mediate immunosuppressive function at tumor sites are not fully elucidated, but increased infiltration of Tregs within the tumor was shown to correlate with worse prognosis in multiple studies (52–55).

As the complexity of CAF populations is being gradually revealed, it is increasingly appreciated that mediating immunosuppression may be operative in a distinct subpopulation of CAFs: FACS-based analysis of CAFs in human breast tumors by using six surface markers identified four distinct CAF subsets which accumulated differently in different subtypes of human breast cancer (luminal A, Her2^+^, and triple-negative). Of these CAF populations, the subtype designated CAF-S1, characterized by expression of FAP, smooth muscle actin α (αSMA), PDGFRβ, and CD29, was found to be associated with recruitment, retention and differentiation of Treg cells: By secreting CXCL12, CAF-S1 promoted the attraction of CD4^+^CD25^+^ T cells, and mediated their retention via expression of OX40L, PD-L2, and Junctional adhesion molecule B (JAM2). Moreover, CAF-S1 were able to increase T cell survival, induce their differentiation to CD4^+^CD25^+^FOXP3^+^ regulatory lymphocytes and to enhance the capacity of Treg cells to inhibit the proliferation of CD4^+^CD25^+^ T cells *in vitro* (20). This mechanism is not restricted to breast cancer, as the presence and function of CAF-S1 in attraction, survival, and differentiation of CD25^+^FOXP3^+^ T lymphocytes was also demonstrated in human high-grade serous ovarian cancers (HGSOC), where it was shown to depend on differential regulation of CXCL12 by miR-200/141. Interestingly, upregulation of FOXP3 required direct cell-cell contact between CAF-S1 and CD4^+^ T cells (56). These findings are consistent with findings from immune competent mouse models which showed that targeting the CXCL12/CXCR4 axis inhibited the recruitment of Treg lymphocytes in ovarian and pancreatic cancer (45, 57).

Thus, by mediating the recruitment of various innate and adaptive immune cells, CAFs shape the immune composition of the TME and support tumor growth (Table 1; Figure 1).

**Table 1:** Recruitment or exclusion of immune cells.

**Effect on immune cells**	**Type of immune cells**	**Cancer type**	**Tumor site**	**Molecule produced by CAFs**	**Targeted**	**References**
Recruitment of myeloid cells	F4/80^+^ macrophages	Breast	Primary	Chi3L1	No	(19)
	THP-1 monocytes	Breast	Primary	IL-6, CCL5 and CCL2	No	(35)
	CD11b^+^Gr1^+^ MDSCs	CRC	Primary	CCL2	No	(39)
	CXCR2^+^ neutrophils	Breast	Primary	CXCL1; CXCL2 and CXCL5	Inhibition of one of CXCR2 ligands	(34)
	CD11b^+^Ly6C^+^ monocytes, and F4/80^+^ macrophages	Lymphoma	Primary	CCL2	No	(33)
	Granulocytic MDSCs (Ly6C^−^Ly6G^+^)	Squamous cell carcinoma	Primary	SASP (CCL8; CXCL5; CCL2; CCL7; IL-6; CXCL1; CXCL14; CCL5)	Depletion of Ly6G^+^ cells	(51)
	CCR2^+^ circulating MDSCs	Hepatic	Primary	CCL2	No	(38)
	Granulocytic MDSCs (Ly6C^−^Ly6G^+^)	Colon, lung, breast, and melanoma	Primary	CXCL1	FAP-CAR T cells	(47)
	CD11b^+^Gr1^int^ F4/80^+^macrophages	PDAC	Primary		FAP Inhibition (UAMC-1110)	(40)
	Monocytes	Prostate	Primary	SDF-1	No	(44)
	CXCR4^+^ mast cells	Prostate	Primary	SDF-1	No	(48)
	Monocytes	Breast	Ex-vivo	CCL2	No	(43)
	CD206^+^ TAMs	Breast		CCL2	Zoledronic acid	(42)
Inhibition of T cell infiltration	CD8^+^ T cells	Breast	Primary	Chi3L1	No	(19)
Inhibition of T cell infiltration & activation	CD3^+^ T cells	Squamous cell carcinoma	Primary	SASP (CCL8; CXCL5; CCL2; CCL7; IL-6; CXCL1; CXCL14; CCL5)	Depletion of Ly6G^+^ cells	(51)
Recruitment and retention of Treg cells	Treg (CD3^+^CD4^+^ FOXP3^+^)	Squamous cell carcinoma	Primary	SASP (CCL8; CXCL5; CCL2; CCL7; IL-6; CXCL1; CXCL14; CCL5)	Depletion of Ly6G^+^ cells	(51)
	CD4^+^CD25^+^ T cells	Breast and HGSOC	Primary	SDF-1; OX40L, PD-L2, and JAM2.	No	(20, 56)
Recruitment of neutrophils	Peripheral blood neutrophils	Hepatocellular carcinoma	Primary	SDF-1	No	(58)

## CAFs Drive an Immunosuppressive Function in Immune Cells

In addition to their capacity to recruit immune cells that foster tumor growth, CAFs were also implicated in affecting the function of various immune cells toward an immunosuppressive phenotype by multiple mechanisms. This may resonate the role of fibroblasts in wound healing, where their function favors M2-like and Th2 type immune reactions (59, 60). Notably, our recent understanding of CAF heterogeneity in origin and function suggests that driving immune suppression may be mediated by distinct subpopulations of CAFs. For example, bone marrow-derived mesenchymal stromal cells (MSCs) were shown to be a significant source of CAFs in breast cancer (12). Interestingly, in physiological wound healing MSCs were shown to mediate immunosuppression: In models of acute liver injury, the presence of pro-inflammatory cytokines (IFNγ, TNFα, or IL-1) induced MSCs to produce iNOS, which in turn suppressed the function of T cells (61, 62). These physiological functions of MSCs and fibroblasts may be hijacked in tumors, to elicit the formation of an immunosuppressive TME via the effect of CAFs on specific immune cell populations (Figure 1).

### Tumor-Associated Macrophages

Tumor-associated macrophages (TAMs) are a heterogeneous cell population arising from circulating monocytes or from tissue resident macrophages, and were implicated in various tumor-promoting tasks including pro-inflammatory signaling, enhancement of angiogenesis, invasion, metastasis and therapy resistance (63, 64). Macrophages can be classified according to their functional differentiation state and immunological responses: M1-like macrophages are involved in the response of type I T helper cells (Th1), they are activated by interferon gamma (IFNγ) and engagement of Toll-like receptors (TLRs) and characterized by production of pro-inflammatory molecules, nitric oxide (NO) and reactive oxygen species (ROS). M2-like macrophages, which are in general pro-tumorigenic, are involved in Th2-type immune responses, wound healing and tissue repair, activated by IL-4 and IL-13, and characterized by promotion of angiogenesis and secretion of immune suppressive factors that inhibit killing by cytotoxic T cells (63). This classification however, is not dichotomous, and different macrophage subtypes may share multiple features.

Functional differentiation of TAMs in the tumor microenvironment is affected by many factors. Recently, CAFs are emerging as novel effector cells in TAM differentiation toward an immunosuppressive phenotype, in addition to their role in monocyte recruitment. CAF-derived Chi3L1 was shown to be important for both recruitment and functional differentiation of bone marrow-derived macrophages in a mouse model of breast cancer: Genetic targeting of Chi3L1 expression in fibroblasts attenuated macrophage recruitment and their reprogramming to an M2-like phenotype, and promoted a Th1 phenotype in the tumor microenvironment (19). Prostate CAFs were shown to mediate both the recruitment and the M2-like differentiation of monocytes via SDF1 (44). A similar finding was demonstrated in an *ex-vivo* model of oral squamous cell carcinoma. CAFs isolated from human tumors, instigated an M2-like phenotype in patient-derived CD14^+^ myeloid cells (manifested by production of ARG1, IL-10 and TGF-β), which in turn potently suppressed the proliferation of autologous T cells (65). However, the underlying CAF-derived factors that mediated M2-like differentiation were not identified.

In this context, it is important to note that while TAMs and CAFs are both central players in the tumor microenvironment, their reciprocal interactions in cancer are not well characterized, and the main focus in the literature is on the effects of macrophages on fibroblasts. Future studies are required to further elucidate the contribution of CAF-derived signaling to the diverse functions of macrophages in the TME (Figure 1).

### MDSCs

Myeloid-derived suppressor cells (MDSCs) are a heterogeneous population of immature immune-suppressive myeloid cells. MDSCs are not found in healthy tissues and appear in pathologic conditions associated with chronic inflammation or stress, as well as in the microenvironment of tumors (29). The functional and phenotypic heterogeneity of MDSCs has been a source of confusion in their definition and terminology (66). Moreover, mouse and human MDSCs may be different in both surface markers (as detailed above) and in their immunosuppressive capacity: while in mice both granulocytic and monocytic MDSCs were shown to inhibit T cells *in vitro*, in human studies findings on the function of granulocytic vs. monocytic MDSCs depend on cancer type, underlying their diversity (30, 67). These differences should be considered when assessing murine studies.

Nevertheless, several studies in mouse and in human experimental systems demonstrated that CAFs are capable of reprogramming an immunosuppressive function in immature myeloid cells, typical of MDSCs. Primary pancreatic stellate cells (PSCs) isolated from human pancreatic tumors, but not normal PSCs, were demonstrated to induce an MDSC phenotype in peripheral blood mononuclear cells (PBMCs), manifested by inhibition of T cell proliferation *in vitro*. This reprogramming was dependent on IL-6 and STAT3 as their inhibition attenuated the induced immunosuppressive function (68). Moreover, IL-6 was found to be predominantly expressed in the stroma of human pancreatic tumors, and targeting it in transplantable and transgenic mouse models of pancreatic ductal adenocarcinoma (PDAC) in combination with PD-L1 blockade resulted in attenuated tumor growth, prolonged survival in a transgenic model of PDAC, and increased presence of intratumoral T cells (69). Importantly, this pathway may not be specific to pancreatic cancer: CAFs isolated from human hepatocellular carcinomas were capable of driving an immunosuppressive gene signature and functions in monocytes and in neutrophils via IL-6 mediated activation of STAT3 signaling (58, 70). Taken together, these findings suggest that combination of immune checkpoint therapeutics with targeting of stromal signaling may be beneficial in treatment of pancreatic and liver carcinomas.

Interestingly, stromal signaling that drives differentiation of peripheral MDSCs may be bone marrow-derived: In transplantable models of lung carcinoma and melanoma (Lewis lung carcinoma and B16 melanoma cell lines), tumor-bearing mice had elevated systemic levels of Dickkopf-related protein 1 (Dkk1), which were found to originate in the bone stromal compartment (osteoblasts and osteocytes) (71). Dkk1 effect on MDSCs was via inhibition of β-catenin, previously shown to be essential in mediating immunosuppressive functions of MDSCs (72). Thus, stromal signaling is central to shaping the functional differentiation of immature myeloid cells toward an immunosuppressive phenotype both locally and systemically in a variety of tumor types.

Importantly, the interactions of CAFs with recruited myeloid cells are reciprocal: Once monocytic and granulocytic cells are recruited to the TME they affect the activation of fibroblasts. For example, activated neutrophils secrete large amounts of reactive oxygen species which are known pro-fibrotic mediators (4, 18). Moreover, activated neutrophils release granules containing multiple proteases including MMPs, elastase, and cathepsins, capable of cleaving collagenous and non-collagenous connective tissue components. This release of ECM breakdown products further activates stromal fibroblasts, physiologically programmed to facilitate matrix remodeling during tissue repair. Similarly, macrophage secreted factors were demonstrated to facilitate reprogramming of resident dermal fibroblasts, or of mesenchymal stromal cells in an NF-κB dependent manner in skin and gastric carcinomas (32, 73).

### T Cells

T cell-mediated immune response can be classified into Th1 or Th2-type immunity, based on their profile of cytokine production. In general, Th2-mediated immunity is considered tumor promoting, as it entails pro-angiogenic signaling, activation of M2-like macrophage function and inhibition of cell-mediated tumor cell killing (74).

Accumulating evidence suggest that signaling by CAFs may shape the T cell milieu in the TME toward a tumor-promoting function, either directly or via innate immune cells. Many of the findings emerge from murine models of cancer in which targeting of specific signaling molecules in CAFs resulted in attenuation of tumor growth and metastasis, accompanied by a shift in the T cell responses: *In vivo* elimination of FAP^+^ CAFs by vaccination lead to a switch from Th2 to Th1-type immunity, characterized by increased expression of the cytotoxic cytokines IL-2 and IL-7, increased CD8^+^ T cell tumor infiltration, and diminished recruitment of macrophages, MDSCs and T regulatory cells in a transplantable model of triple-negative breast cancer (75). Targeting of CAF-derived Chi3L1 had a similar effect in another transplantable model of breast cancer, and resulted in enhanced infiltration of CD8^+^ T cells and a shift in the tumor cytokine profile toward a Th1-type phenotype. However, in both studies these effects of CAFs on T cells may be indirect.

A direct effect of CAFs on T cell function was demonstrated in an *in vitro* study that utilized fibroblasts and tumor-infiltrating T lymphocytes (TILs) isolated from human lung tumors: CAF-derived IL-6 enhanced production of IFNγ and IL-17A in activated TILs, suggesting that fibroblasts may also have an immunostimulatory effect on T cells (76).

Notably, the crosstalk between CAFs and T cells is reciprocal. Secreted factors from activated T cells enhanced the production of IL-6 by lung CAFs (76). Activated lymphocytes were also shown to induce the expression of cyclooxygenase-2 (COX-2) and intercellular adhesion molecule-1 (ICAM-1) in normal human lung fibroblasts. These activated fibroblasts then induced a reduction in the expression of T cell activation/co-stimulation markers (CD69, LFA-1; CD3 and CD28) suggesting that fibroblasts are able to modulate effector functions of T cells recruited into sites of inflammation (77) (Figure 1).

Many of the pathways that are operative in tumors represent “hijacking” of physiological pathways. Indeed, the interactions between fibroblasts and T cells are probably not restricted to tumors and represent a physiological role of fibroblasts, as normal skin fibroblasts and autologous T cells showed similar interactions *in vitro* (76). The ability of fibroblasts to drive type-2 immunity may also be a physiological capability of fibroblasts: A recent study in normal lung tissue suggested that fibroblast-like adventitial stromal cells (ASCs) support the accumulation and activation of group 2 innate lymphoid cells (ILC2s), which are important mediators of type 2 immunity. Accumulation of ILC2s resulted in a formation of a tissue niche with Treg and dendritic cells, and depletion of ASCs abrogated these functions, suggesting that subpopulations of fibroblasts are required for optimal accumulation of ILC2s during type 2 immune responses (78). Future studies are required to demonstrate whether these fibroblast-mediated functions are also operative in tumors.

### Dendritic Cells

Another mechanism by which CAFs hinder anti-tumor immune responses and impede on the function of T cells in the TME is by affecting the function of DCs, the most important population of antigen-presenting cells. Activated fibroblasts are a major source of transforming growth factor b (TGF-β), a pleotropic and immunosuppressive cytokine that functions in wound healing, ECM remodeling, and can affect the functional differentiation of multiple types of immune cells (79). TGF-β was shown to mediate downregulation of MHC class II molecules and the co-stimulatory molecules CD40, CD80, and CD86 in dendritic cells, thus inhibiting their antigen presentation capacity and their capability to activate cytotoxic T cell responses (80). CAF-mediated modulation of DC function was also shown to be mediated by their secretion of pro-inflammatory cytokines (Figure 1). CAFs isolated from human hepatic carcinomas were shown to secrete IL-6, which activated STAT3 in DCs, resulting in generation of regulatory DCs. These CAF-educated DCs exhibited lower expression of antigen presenting molecules and co-stimulatory molecules (CD1a, HLA-DR, CD80, CD86), and elevated expression of immunosuppressive cytokines (such as IL-10 and TGF-β). Moreover, hepatic CAF-educated DCs could affect T cells toward a suppressive phenotype, including induction of CD4^+^CD25^+^Foxp3^+^ Tregs, and decreased production of IFN-γ in CD8^+^ T cells (81). In lung cancer, inhibition of DCs differentiation and function was shown to be mediated via CAF-secreted tryptophan 2,3-dioxygenase (TDO2). Analysis of lung cancer surgical specimens revealed increased TDO2 expression in the fibroblasts adjacent to the cancer, and inhibition of TDO2 in a transplantable model of lung carcinoma resulted in improved DC function and T cell response, and decreased experimental metastasis (82).

The crosstalk between CAFs and dendritic cells was also shown to affect the ability of DCs to polarize the differentiation of T cells toward a Th2 phenotype in pancreatic cancer, via CAF secretion of Thymic stromal lymphopoietin (TSLP). CAFs that were activated by tumor-derived pro-inflammatory signaling (TNFα and IL-1β) secreted TSLP, which endowed them with the ability to drive the differentiation of naïve CD4^+^CD45RA^+^ T cells toward a Th2 phenotype. Human data from pancreatic cancer patients indicated that DCs with features of TSLP-treated DCs and Th2-attracting chemokines were present in pancreatic tumors, and the Th2/Th1 ratio in pancreatic tumors was an independent marker of poor survival (83).

Taken together, these studies demonstrate multiple mechanisms by which CAFs modulate the functional differentiation of immune cells in the TME toward an immunosuppressive function (Table 2, Figure 1).

**Table 2:** CAF-mediated modulation of immune cell differentiation.

**Effect on immune cells**	**Type of immune cells**	**Cancer type**	**Tumor site**	**Molecule produced by CAFs**	**Targeted**	**References**
M2-like differentiation	Circulating monocytes	Prostate	Primary	SDF-1	No	(44)
	TAMs	Breast	Primary	Chi3L1	No	(19)
Inhibition of Th1 immunity	Th1/Th2 cells	Breast	Primary and lung metastases	Not specified	Elimination of CAFs via pFAP vaccination	(75)
Th17 Differentiation	T cells (Th17 polarization)	Lung	Primary	IL-6	No	(76)
Shaping the activity of dendritic cells	Th2 polarization via DC conditioning	Pancreatic	Primary	TSLP	No	(83)
	DC	Hepatocellular carcinoma	Primary	IL-6	No	(81)
	DC	Lung	Primary	Kyn	TDO2 inhibitor	(82)
MDSCs differentiation & Activation	Monocytes	Hepatocellular carcinoma	Primary	SDF-1	No	(70)
	MDSCs	Melanoma and lung adeno-carcinoma	Primary	Dkk1	Inhibition of Dkk1	(71)
	Peripheral blood mononuclear cells	Pancreatic	Primary	IL-6, VEGF, M-CSF, SDF-1, MCP-1	IL-6 neutralization	(68, 69)
Treg cell Differentiation	CD4^+^CD25^+^ FOXP3^+^ Treg	Breast and HGSOC	Primary	B7H3, CD73, DPP4	No	(20, 56)

### CAF-mediated ECM Remodeling and Fibrosis Drives an Immunosuppressive Microenvironment

Fibrosis is a scarring process, characterized by excess deposition of collagenous and non-collagenous extracellular matrix (ECM) due to the accumulation, proliferation, and activation of fibroblasts. One of the hallmarks of CAFs is the excessive production/deposition of extracellular matrix components and degradation enzymes. This CAF-mediated deregulation of the ECM results in biomechanical and biochemical changes that facilitate tumor growth, invasion, and metastasis (84, 85). In addition to their effect on cancer cells, CAF-mediated deregulation of the ECM protein network modulates immune cells trafficking. Aberrant ECM protein composition and fragments of the ECM that are derived from tissue-remodeling processes can influence immune cell activation and survival, thereby actively contributing to immune responses at these sites (86). Various ECM components were shown to modulate macrophage polarization (toward an M2-like signature) and mediate the migration and maturation of monocytes and MDSCs (87). Moreover, stiffed collagen-rich matrix was found to induce CAFs production of monocytic chemoattractants like CCL2 and M-CSF (87), and CAFs in tumor-associated fibrosis produce high levels of cytokines and chemokines that favor tumor-promoting Th2 and Th17 responses (88). One example for an ECM component demonstrated to affect macrophage trafficking to tumors is hyaluronan (HA). Genetic targeting of the HA synthase gene in fibroblasts in a transplantable model of mammary carcinoma, leading to HA deficiency in the stroma, resulted in impaired macrophage recruitment and attenuated tumor angiogenesis and lymphangiogenesis (89).

While ECM components promote immune cell recruitment and activation, excessive deposition of collagen by fibroblasts in the TME leading to formation of scar-like tissue, was shown in pancreatic cancer to form a physical barrier that prevented cytotoxic T cell infiltration into the tumor, thus contributing to immune escape in pancreatic cancer. *In vitro* experiments demonstrated that while activated T cells migrated in low-density collagen matrices, migration was inhibited in dense collagen (90). Real-time imaging in viable slices of human lung tumors revealed that antigen-specific T cells within the tumor accumulate more in the stromal rich area than in the tumor islets. The density and the orientation of collagen and fibronectin fibers were suggested to play key roles in controlling T cells trafficking, as matrix loosening induced by collagenase treatment increased the ability of T cells to contact tumor cells (91, 92). Indeed, highly desmoplastic stroma, associated with activation of focal adhesion kinase (FAK) in pancreatic tumors, was shown to correlate with poor CD8^+^ cytotoxic T cell infiltration. FAK inhibition in a transgenic mouse model of PDAC resulted in attenuated tumor fibrosis, improved response to immune checkpoint therapy and prolonged survival. These findings suggest that targeting fibrosis may be beneficial for overcoming CAF-mediated immune suppression (93).

The composition of the ECM is an important factor in enabling tumor metastasis (94). CAF-mediated remodeling of the ECM was recently shown to have an important role in enabling melanoma metastasis, in association with aging. While young skin fibroblasts produced abundant ECM components, aged fibroblasts were shown to lose the expression of the hyaluronic and proteoglycan link protein (HAPLN1), resulting in enhanced alignment of ECM matrices that promoted metastasis of melanoma cells in a mouse model of transplantable melanoma. However, the effect of matrices produced by aged fibroblasts was inhibitory on the migration of T cells, which may contribute to impaired immune response in the TME (95). Interestingly, age-related changes in HAPLN1 were also shown to increase lymphatic permeability, which affected melanoma lymph node metastasis. Age-related loss of HAPLN1 was shown to be associated with loss of integrity in the lymphatic vasculature and with enhanced lymphatic endothelial permeability, which enabled the escape of melanoma cells from the lymphatic system to distant metastatic sites (96).

Thus, CAF-mediated ECM remodeling and fibrosis contribute to the formation of an immunosuppressed and growth promoting microenvironment by multiple mechanisms (Figure 1).

## Direct Inhibition of Cell Mediated Killing: CAFs Abrogate the Function of Cytotoxic Lymphocytes

In addition to mediating the recruitment and functional differentiation of immune cells in the TME, findings from multiple studies implicate CAFs in affecting killing of tumor cells by cytotoxic lymphocytes.

CAFs isolated from human metastatic melanoma lesions or from hepatocellular carcinomas interfered in co-culture experiments with NK ability to kill melanoma cells. This inhibition was mediated by CAF-derived PGE2 and IDO, which abrogated NK cells expression of cytotoxic molecules (granzyme B and perforin) and cytokines, and impaired their cytotoxic activation and surface expression of NKp44 and NKp30 (97, 98). Another mechanism by which CAFs abrogate NK killing of tumor cells was demonstrated in melanoma-associated fibroblasts: CAFs isolated from tumors of melanoma patients decreased *in vitro* susceptibility of cancer cells to NK-mediated lysis. Mechanistically, CAFs secreted active matrix-metalloproteinases (MMPs) that were able to degrade two NKG2D ligands (MICA/B) on the surface of melanoma cells, resulting in an inhibition of NKG2-dependent cytotoxic activity of NK cells (99).

CAFs were also shown to directly abrogate the function of cytotoxic T cells by multiple mechanisms: Expression of immune checkpoint molecules is emerging as an important process by which CAFs directly suppress the function of cytotoxic T lymphocytes (CTL). Fibroblasts isolated from melanoma patient biopsies could directly suppress CD8^+^ T cells proliferation and function, via upregulating their expression of the PD-1 ligand PD-L1, mediated by IL-1α/β. These finding suggest that blockade of IL-1 may benefit melanoma patients and potentially synergize with immunotherapeutic interventions (100). CAFs isolated from resected human pancreatic tumors were recently shown to express PD-L1 and PD-L2. Furthermore, these CAFs were shown *in vitro* to inhibit the proliferation of T cells and to stimulate their expression of the inhibitory molecules TIM-3, PD-1, cytotoxic T-lymphocyte-associated protein 4 (CTLA-4) and LAG-3, possibly via the activity of CAF-derived PGE2 (101).

Expression of immune checkpoint molecules is likely a physiological pathway in fibroblasts during inflammation: Normal colon fibroblasts expressing PD-L1 and/or PD-L2 were shown to be involved in the regulation of mucosal CD4^+^ T cell response (102). Moreover, expression of PD-L1 is upregulated in human dermal fibroblasts and in mesenchymal stromal cells via IFNγ, highly secreted by activated T cells (103, 104). Future studies are required to assess whether IFNγ also mediates the expression of PD-L1/2 in CAFs in tumors.

Another suggested mechanism for abrogating the function of cytotoxic T cells is by CAF-mediated metabolic effects. MSCs isolated from cervical tumors had elevated expression levels of CD39 and CD73 as compared with normal tissue fibroblasts. This feature was associated with the ability to strongly suppress the proliferation, activation and effector functions of cytotoxic T cells through generation of large amounts of adenosine from the hydrolysis of ATP, ADP, and AMP nucleotides (105). Similarly, glycolytic CAFs in prostate cancer were found to affect the polarization and function of effector T cells via their release of lactate (106).

Immunotherapy approaches are designed to unleash the cytotoxic T cell function of “dysfunctional” CD8^+^ T cells by blocking the immunosuppressive signaling restraining these T cells. This requires not only that activated cancer-specific T cells be present in the TME, but also that their location allows physical contact with tumor cells. Many tumors exhibit an “immune excluded” phenotype, in which T cells are restricted to a peritumoral zone rich in fibroblasts, with few lymphocytes within the epithelial tumor mass itself (107). In a murine model of pancreatic cancer, CAF-derived CXCL12 protected tumor cells from T cell accumulation. Depletion of FAP^+^ CAFs resulted in enhanced T cell infiltration and better response to anti-CTLA4 and anti-PD-L1 treatment in mice (45, 92, 108).

Indeed, CAFs were suggested to be important in affecting the non-effectiveness of immune therapy in multiple cancer types, partially via activation of TGF-β signaling. TGF-β signature in fibroblasts was shown to be associated with poor response to anti-PD-L1 treatment in metastatic urothelial cancer. Moreover, TGF-β signaling in fibroblasts was correlated with exclusion of CD8^+^ T cells within the tumor, which were instead found in the fibroblasts and collagen-rich peritumoral stroma. Therapeutic co-administration of TGF-β-blocking reagents and anti-PD-L1 antibodies reduced TGF-β signaling in stromal cells, facilitated T-cell tumor penetration, and promoted tumor regression (109).

Similarly, targeting of TGF-β in a transgenic mouse model of metastatic colorectal cancer unleashed a potent and enduring cytotoxic T cell response. Tumors in these mice were characterized by T cell exclusion, highly activated stromal TGF-β, and a limited response to anti-PD-1 and anti-PD-L1 treatment. Inhibition of TGF-β enabled T cell infiltration to tumors and metastatic lesions, facilitated the response to anti-PD-1 and anti-PD-L1 therapy, diminished tumor growth and reduced liver metastasis (110). Taken together, these studies suggest that CAFs play a central role in inhibiting tumor cell killing by T cells, and advocate the use of combination therapies that target immune checkpoint inhibitors together with abrogating the immunosuppressive ability of CAFs (111).

Strikingly, recent evidence suggested that CAFs are capable of antigen presentation, leading to antigen-specific deletion of CD8^+^ T cells to protect tumor cells. CAFs isolated from lung adenocarcinomas and melanoma tumors were shown to process and present antigens, and directly interact with activated CD8^+^ T cells, thus inducing T cell death via PD-L2 and FAS ligand (FASL) engagement. Moreover, the capacity of antigen-specific T cells to kill their target tumor cells was dramatically impaired when conditioned by antigen-loaded CAFs, indicating that CAFs are capable of driving dysfunction and death of tumor-specific T cells, leading to enhanced tumor cell survival (112).

Interestingly, this function of CAFs is similar to the physiological role of lymph node fibroblastic reticular cells (FRCs) that display specific immunological properties to maintain peripheral tolerance. Lymph node structure consists of defined niches for B and T lymphocytes. This structure is provided by FRCs, which also provide the lymphocytes with a scaffold upon which to migrate. FRCs produce collagen rich reticular fibers that form a dense network within the lymphoid tissue. The network of fibers supports and guides the movement of dendritic cells (DCs), T lymphocytes and B lymphocytes (113). Moreover, FRCs express and present peripheral tissue antigens to T cells in lymph nodes (LNs), receive peptide-MHC II loaded exosomes from DCs, induce CD4^+^ T cell hyporesponsiveness, and dampen T cell proliferation through the production of nitric oxide (114). These physiological functions may be hijacked in tumors, granting CAFs the capacity to actively regulate T cell function within the TME. For example, in lymph nodes, T cell migration is guided by FRCs that secrete the CCR7 ligands CCL21 and CCL19, which guide the interactions between CCR7^+^ T cells and antigen-presenting cells (APCs) needed for T cell education and priming (115). These events are central for maintaining peripheral tolerance, as Treg cells require LN occupancy and CCR7 signaling for their activation and function, and the loss of CCR7 signaling is associated with spontaneous autoimmunity. Interestingly, similar pathways of immune tolerance and T cell exclusion are operative in tumors: Expression of CCL21 in melanoma tumors in mice was associated with the induction of stromal zones that were reminiscent of lymph node paracortex stroma, recruitment of regulatory immune cells, an altered cytokine milieu, and an immunotolerant microenvironment, which depended on host expression of CCR7 (116).

In human and mouse breast tumors, this direct immunosuppressive capacity of CAFs was attributed to a distinct subpopulation of fibroblasts expressing FAP and Podoplanin (PDPN). FAP^+^PDPN^+^ CAFs expressed a TGF-β and fibrosis-related gene signature, and were in direct contact with T cells in the peritumoral dense ECM of mammary tumors. Moreover, FAP^+^PDPN^+^ CAFs were shown to suppress T cell proliferation in a nitric oxide-dependent manner (117). This function is reminiscent of the immune suppressive function of FRCs in the lymph nodes: Under inflammatory conditions, FRCs acquire immunosuppressive potential, and attenuate T cell expansion by producing nitric oxide (118, 119).

Thus, tumor cells co-opt tissue fibroblasts to generate stromal architecture and function that restrains tumor-infiltrating immune cells and impedes proper function of cytotoxic lymphocytes (Table 3, Figure 1). Taken together, these studies suggest that better understanding of CAF interactions with T cells and with regulation of immune checkpoint pathways may be beneficial for better design of immunotherapy treatments.

**Table 3:** CAF-mediated inhibition of anti-tumor cytotoxicity.

**Effect on immune cells**	**Type of immune cell**	**Cancer type**	**Tumor site**	**Molecules produced by CAFs**	**Targeted**	**References**
Inhibiting NK cytotoxic activities	NK cells	Melanoma	Metastatic lesions	PGE2	No	(97)
		Hepatocellular carcinoma	Primary	PGE2 and IDO	No	(98)
Exclusion of CD8^+^ T cells	T cells	Pancreatic	Primary	CXCL12	Depletion of FAP^+^ cells	(45, 92, 108)
		Urothelial cancer	Metastatic lesions	TGF-β	No	(109)
Inhibition of T cell activity		Pancreatic	Primary	PD-L1,2 and COX-2	PGE2 inhibitor	(110)
		Colorectal	Primary	TGF-β	TGF-β inhibitor	(110)
Exclusion and killing of CD8^+^ T cells		Lung adeno-carcinoma and melanoma	Primary	FAS-L and PD-L2	No	(112)
Suppression of proliferationand activation		Breast	Primary	FAP and PDPN; TGF-β	No	(117)
		Cervical cancer	Primary	CD39 and CD73	No	(105)

## Therapeutic Perspectives: CAF Targeting Approaches

Therapeutic approaches of treating cancer are increasingly moving toward combinatorial strategies that target key operative pathways and mediators in the TME, rather than solely targeting cancer cell-intrinsic pathways. This is a result of improved understanding of the complexity of tumor eco-systems, as well as improved capacity of precision diagnostics that enable tailored therapeutic approaches. As our understanding of the important role of CAFs in mediating multiple tumor-promoting functions increases, it becomes clear that targeting CAFs in combination with other therapeutics may be beneficial. Based on the role of CAFs in mediating an immunosuppressed microenvironment that was reviewed herein, co-targeting of CAFs in combination with immunotherapeutics is an attractive option.

Pre-clinical trials targeting CAFs indicated that targeting a subpopulation of FAP^+^ CAFs was beneficial in transplantable models of Lewis lung carcinoma and pancreatic adenocarcinomas. Depletion of FAP^+^ CAFs using transgenic mice with FAP promoter- driven diphtheria toxin receptor (DTR) resulted in tumor regression in an IFNγ and TNFα dependent manner (120). Moreover, depletion of FAP^+^ CAFs in a mouse model of PDAC enabled the therapeutic effects of anti-CTLA4 and anti-PD-1 (45). In an effort to design more applicable ways to target FAP, multiple other approaches were developed for the targeting of FAP^+^ CAFs, including pharmacological inhibitors (e.g., PT630) (121), monoclonal antibodies (FAP5-DM1) (122), a FAP- targeting immunotoxin (αFAP- PE38) (123), and an oral DNA FAP vaccine (124, 125), which showed efficacy in mouse models of breast, pancreatic, lung and colon carcinomas. Moreover, a chimeric antigen receptor (CAR) T-cell specific for FAP was demonstrated to inhibit the growth of various subcutaneously transplanted tumors in mice by augmenting CD8^+^ T cell antitumor responses (126). However, depletion of FAP^+^ cells using the DTR system had severe systemic toxicity, including cachexia and anemia in mouse models of transgenic PDAC (KPC mice) and in transplantable colon carcinoma (C26 cells), likely reflecting the importance of FAP^+^ stromal cells in maintaining normal muscle mass and hematopoiesis (127). Thus, caution should be taken when designing FAP-targeting approaches for clinical testing in cancer patients.

Depletion of αSMA^+^ myofibroblasts in a mouse model of PDAC, utilizing thymidine kinase-Ganciclovir-mediated ablation, resulted unexpectedly in more invasive tumors, increased presence of CD4^+^Foxp3^+^ Treg cells and reduced survival (128). These findings suggest that the effect of targeting CAFs may depend on tumor type and on the experimental systems that were used, and requires careful consideration. In this context, it is important to note that depleting entire fibroblast populations is highly problematic in human patients, as fibroblasts have many critical physiologic functions. Moreover, both αSMA and FAP are not expressed exclusively by CAFs, which adds to the complexity of targeting cell populations based on these markers.

Therefore, targeting of molecules or pathways that are essential for the tumor-promoting functions of CAFs is likely a more clinically relevant approach. For example, targeting the pro-fibrotic function of CAFs with Pirfenidone (PFD—an anti-fibrotic agent as well as a TGF–β antagonist) was shown to be efficient in combination with doxorubicin in a mouse model of triple-negative breast cancer (4T1) (129). Similarly, targeting the fibrotic activity of CAFs with tranilast, an anti-fibrotic agent, in transplantable tumor models (lymphoma, Lewis lung carcinomas and melanoma) resulted in decreased presence of Treg cells and MDSCs, and enhanced cytotoxic CD8^+^ T cell response. These beneficial tumor-inhibitory effects were enhanced when CAFs were targeted in combination with effector-stimulatory immunotherapy such as dendritic cell-based vaccines (130). Importantly, CAF-mediated fibrosis contributes not only to enhanced tumor growth and invasiveness, but also to the immunosuppressive role of CAFs, as the increased matrix stiffness forms a physical barrier that limits the access of anti-tumor immune cells (87). Thus, targeting the pro-fibrotic functions of CAFs is beneficial for multiple reasons.

Another attractive option is targeting the transcriptional reprogramming of CAFs, which contributes to their activation. Vitamin D receptor (VDR) in pancreatic stellate cells was shown to be a central transcriptional repressor of their inflammatory and fibrotic functions, and treatment of mice with the VDR ligand calcipotriol induced stromal reprogramming that inhibited inflammation and fibrosis, enabled gemcitabine delivery into tumors, and improved survival in a PDAC model, suggesting that vitamin D may be utilized therapeutically in the treatment of pancreatic cancer (131). In addition, targeting central cytokines and chemokines that contribute to the pro-inflammatory, immunosuppressive and matrix remodeling function of CAFs may also be beneficial. For example, targeting IL-6 was suggested as a stromal-targeting therapeutic approach in cancer (132). In addition, CAFs are the main source of SDF-1/CXCL12 and blockade of the SDF-1/CXCL12-CXCR4 signaling pathway was shown to be beneficial in alleviating CAF-mediated immunosuppression (45, 92).

Importantly, the functional complexity and heterogeneity of CAF populations that may be specific to tumor type, specific organ and physiological context warrants careful consideration of CAF-targeted therapeutic strategies in patients.

## Future Challenges and Outlook

The central role of fibroblasts in all stages of tumorigenesis and metastasis has emerged in recent years, as part of our growing understanding of tumors as multicellular organs. In addition to their “classical” functions in matrix remodeling and secretion of ECM components, accumulating evidence from many studies implicate CAFs in immunoregulatory functions that shape the immune milieu of tumors toward a pro-inflammatory and immunosuppressive function. These functions are mediated by CAF secretion of multiple cytokines and chemokines, and by reciprocal interactions with innate and adaptive immune cells. However, the heterogeneity and plasticity of CAFs are still poorly understood. This is partially a result of limited experimental tools: much of our knowledge relies on *in vitro* studies, or studies of CAF co-injection with tumor cells, which may not faithfully recapitulate the physiological function of CAFs. A major limitation to our ability to elucidate the role of specific CAF-derived factors is the sparsity of reliable CAF-specific Cre mice which will enable conditional ablation of candidate factors in CAFs, in order to identify potential therapeutic targets.

Another future challenge is the use of reliable pre-clinical models of spontaneous metastasis (133) that will enable better understanding of the role of CAFs in the formation of a pre-metastatic niche, and in facilitating the early stages of metastasis.

In the coming years, we will likely see multiple studies that will profile CAF populations at the single cell level, enabling better identification of their functional heterogeneity. Such understanding will provide us with both context-specific understanding of unique CAF functions, and unifying mechanisms that are common to CAF tasks in various cancer types. While knowledge from preclinical studies on immunoregulatory functions of CAFs is emerging, clinical data on CAF targeting is still limited. Hopefully, we will see in the future integration of the preclinical findings described in this review (Figure 1) into the design of novel therapeutic combination strategies aimed at impairing the tumor-supportive and immunosuppressive responses of CAFs.

## Author Contributions

LM analyzed the relevant literature, prepared the tables and figures, and participated in writing the manuscript. NE wrote the manuscript.

### Conflict of Interest Statement

The authors declare that the research was conducted in the absence of any commercial or financial relationships that could be construed as a potential conflict of interest.
